# MFN2-a multifaceted guardian against Parkinson’s pathophysiology: mitochondria, ferroptosis, inflammation and oxidative stress

**DOI:** 10.3389/fnagi.2025.1611958

**Published:** 2025-09-16

**Authors:** Yan Cheng, Hongjiang Zhai, Yong Liu, Yunzhou Yang, Bo Fang, Mingxiang Song, Xiuqin Wang, Ping Zhong

**Affiliations:** ^1^Department of Neurology, Lu'an Hospital of Anhui Medical University, Lu'an, China; ^2^Department of Neurology, Suzhou Hospital of Anhui Medical University, Suzhou, China

**Keywords:** Parkinson’s disease, mitofusin 2, ferroptosis, inflammatory, oxidative stress, mitochondrial function

## Abstract

**Background:**

Parkinson’s disease (PD) is the second most prevalent neurodegenerative disease worldwide and its exact pathogenesis remains unclear. This study aims to comprehensively explore the role of MFN2 in PD based on *in vivo* and *in vitro* models for multidimensional understanding.

**Methods:**

*In vivo*, C57BL/6 J male mice were administered MPTP and probenecid by intraperitoneal injection to establish PD models. Lentivirus carrying MFN2 was microinjected into the bilateral striatum of specific groups of mice. The motor and cognitive functions of the mice were evaluated using the rotarod test and the open field test. *In vitro*, SH-SY5Y cells were treated with MPP^+^ to establish cell-based PD models. Transfection of plasmids was used to achieve overexpression or knockdown of MFN2. Subsequently, a series of experiments such as qRT-PCR, Western blot, CCK-8, flow cytometry and ELISA were used to verify the potential mechanism of MFN2.

**Results:**

In PD models, the expressions of DHODH, MFN1, MFN2, GPX4, and FSP1 were significantly down-regulated, and their motor coordination, self-cognitive behavior, and exploration ability were decreased. Concurrently, inflammatory and oxidative stress responses were enhanced, cell viability was weakened, apoptosis was increased, and mitochondrial abnormalities were observed. Overexpression of MFN2 improved the motor, cognitive and neurological damage in mice, enhanced cell viability, inhibited apoptosis, reduced the levels of inflammatory and oxidative stress factors, and up-regulated the expressions of DHODH, MFN1, GPX4 and FSP1. Mitochondrial morphological observation showed that MFN2 overexpression alleviated mitochondrial abnormalities.

**Conclusion:**

MFN2 may play a protective role in PD by regulating mitochondrial function, ferroptosis, inflammation and oxidative stress-related factors, providing a new theoretical basis and potential therapeutic targets for the treatment of PD.

## 1 Introduction

Globally, Parkinson’s disease (PD) is the second most common neurodegenerative disease. It is the fastest growing neurodegenerative disorder, with a global prevalence exceeding 6 million people (Tolosa et al., 2021; Langeskov-Christensen et al., 2024). Despite extensive research, the exact pathogenesis of PD is still elusive. However, it is generally considered to be the result of a complex interaction of genetic, environmental and aging factors (Bloem et al., 2021). To date, available treatments mainly focus on alleviating symptoms rather than halting the disease progression, highlighting the urgent need for ongoing in-depth research into the underlying mechanisms, as this could help develop new ways to treat the disease.

Mitochondrial dysfunction is one of the major causes of PD (Rocha et al., 2018). Mitochondrial dysfunction can trigger a cascade of events, including oxidative stress (Subramaniam and Chesselet, 2013), neuroinflammation (Borsche et al., 2021), and ferroptosis (Wang et al., 2025). Mitofusins, including MFN1 and MFN2, are GTPases localized on the outer mitochondrial membrane. They serve as major regulators of mitochondrial fusion, a process critical for maintaining mitochondrial network integrity and function (Grel and Woznica, 2023). Apart from its role in mitochondrial fusion, MFN2 is also critical for the establishment of interactions between mitochondria and the endoplasmic reticulum (ER), and dysfunction of mitochondria and ER is the basis of metabolic changes (Tur et al., 2020). Studies have indicated that MFN2 exhibits stronger tethering activity compared to MFN1, and this could be associated with its function in maintaining mitochondrial-ER interactions (Li and Cao, 2019; Yapa et al., 2021). As a central modulator of mitochondrial dynamics and interorganelle communication, MFN2 is closely linked to the regulation of mitochondria damage-driven alterations (Jiang et al., 2018; Chen et al., 2023; Wei et al., 2024). Moreover, MFN2 has been implicated in neuronal function and peripheral neuropathy, further supporting its relevance to neurobiological processes (Schneeberger et al., 2013; Tur et al., 2020). In recent years, MFN2 has garnered increasing attention in PD research (Zhao et al., 2017; Wang et al., 2024). However, the precise mechanisms by which MFN2 regulates these mitochondria-associated pathological processes in the context of PD remain unclear. Thus, this study was designed to elucidate the role of MFN2 in PD using integrated *in vitro* and *in vivo* models. Specifically, we explored the effects of MFN2 on mitochondrial function, ferroptosis, inflammation, and oxidative stress-related factors. Additionally, the effects of MFN2 on cell viability, apoptosis, motor, cognitive, and neurological damage in mice, and mitochondrial morphology were also evaluated. Collectively, our results demonstrate that MFN2 may exert a protective effect in PD by modulating mitochondrial function and mitigating mitochondria damage-driven alterations (including ferroptosis, inflammation, and oxidative stress). This study may provide new insights into the role of MFN2 in PD and provide a new theoretical basis and potential therapeutic targets for the treatment of PD.

## 2 Materials and methods

### 2.1 Animal models

C57BL/6 J male mice (7 weeks), purchased from Shanghai Bikai Laboratory Animal Co., LTD, were used in this study. The feeding environment of mice was indoor temperature 20–22 °C, relative humidity 50–60%, free feeding, natural light, and regular cleaning and disinfection. The mice were divided into two parts, one part of 10 were divided into control group and PD model group (5 mice in each group), and the other part of 24 were divided into control group, PD model group, PD model + overexpression group and PD model + overexpression empty vector group (6 mice in each group). After 1 week of adaptive feeding, the mice were modeled. The models were established by intraperitoneal injection of MPTP (20 mg/kg) (23007–85-4, selleck, USA), and then intraperitoneal injection of probenecid (250 mg/kg) (S24877-5 g, yuanye, China) after 1 h. MPTP and probenecid were injected twice every 7 days, and the average interval was as far as possible. The intervention lasted for 5 weeks, with a total of 10 injections. The control group was injected with the same amount of normal saline. In addition, for the mice in the PD model + overexpression and PD model + overexpression empty vector groups, 50 μL of lentivirus was microinjected into the bilateral striatum using brain stereotaxic apparatus on the second day of the first injection of MPTP. The vector used for mouse MFN2 overexpression was GL214 pcSLenti-EF1-EGFP-CMV-MCS-3xFLAG-WPRE. After constructing the overexpression vector, the sequencing primers used were the forward primer 5′-GCTGGACAAGAAGCTGAAGG-3′ and the reverse primer 5′-GGCTGGTAGTCATCCTTGGA-3′.

### 2.2 Rotarod test and open field test

The rotarod test and open field test were performed at the 6th week after MPTP and probenecid injection. The rotarod test was used to detect the muscle strength and motor coordination ability of mice. The mice were placed on the running rotarod roller with a diameter of 3 cm, and the rotation speed was 20 r/min. After the mice adapted twice, the experiment began. The fall latency of mice on the roller was recorded. Each test interval was 3 min, and each mouse was tested 3 times, and the average value was used for statistical analysis. The open field test was used to explore the self-cognitive behavior and exploration ability of mice. After the mice were placed in the intermediate adaptation activity for 10 min, the video behavioral analysis system was used to record the activity time and activity distance of the mice in the central area within 5 min. After each mouse was tested, the feces on the bottom were cleaned and disinfected with 75% alcohol to dry, and then the next mouse was tested. Each mouse was tested 3 times, and the average value was used for statistical analysis.

### 2.3 Sample preparation

After routine anesthesia, blood samples were collected from mice using the decapitation method. At the same time, the whole brain tissue of mice was quickly collected. For the tests based on fresh tissue samples, the ventral midbrain tissues were immediately isolated after simple washing with phosphate buffered saline (PBS). For the samples used for pathological examination under light microscope, the brain tissue samples should be fixed in 4% paraformaldehyde solution for 24 h, and then the ventral midbrain tissue should be separated. For the samples used for electron microscopy detection, the ventral midbrain tissue should be carefully peeled off on the ice surface and fixed in 4% glutaraldehyde solution at 4 °C overnight. For the samples used for the detection of tissue cytokines and protein levels, the ventral midbrain tissues were separated, washed and divided, then plunged into liquid nitrogen for rapid freezing, and subsequently transferred to an −80 °C refrigerator for storage. SH-SY5Y cells were purchased from iCell Bioscience Inc. and used for subsequent experiments after cell recovery and passage.

### 2.4 Quantitative real-time polymerase chain reaction (qRT-PCR)

To verify the expression of target genes at the RNA level, qRT-PCR was performed. The TRIzon Reagent kit (CW0580S, CWBIO, China) was used to extract total RNA according to the manufacturer’s protocol. The HiFiScript cDNA synthesis kit (CW2569M, CWBIO, China) was used for reverse transcription. The UltraSYBR Mixture (Low ROX) kit (CW2601H, CWBIO, China) was used for qRT-PCR. All primer sequences were shown in Supplementary Table S1. Reaction conditions: 95 °C for 10 min, followed by 40 cycles of 95 °C for 15 s and 60 °C for 30 s. The relative quantitative analysis of data was carried out by 2^-△△CT^ method. Each sample was run in triplicate, and experiments were independently repeated at least 3 times.

### 2.5 Western blot

To verify the expression of target genes at the protein level, western blot was performed. Total proteins were extracted using radio immunoprecipitation assay (RIPA) lysis buffe (P0013B, Beyotime, China) containing phenylmethylsulfonyl fluoride (PMSF) at a final concentration of 1 mM. The protein concentration of the sample was measured using Nano300 protein concentration meter. Subsequently, the protein was separated by sodium dodecyl sulfate polyacrylamide gel electrophoresis (SDS-PAGE). The protein bands separated on the gel were transferred to polyvinylidene fluoride (PVDF) membrane (IPVH00010, Millipore, USA) by transfer electrophoresis. Prior to use, the PVDF membrane was activated with methanol for 3 min. The membrane transfer was performed at a constant current of 300 mA, with a transfer duration of 2–3 h. PVDF membranes were incubated with primary antibodies, including DHODH antibody (14877-1-AP, Proteintech, USA), MFN1 antibody (13798-1-AP, Proteintech, USA), MFN2 antibody (12186-1-AP, Proteintech, USA), GPX4 antibody (ab252833, abcam, UK) and FSP1 antibody (20886-1-AP, Proteintech, USA), overnight at 4 °C, and then incubated with the corresponding secondary antibody (A21020 or A21010, Abbkine, China) at room temperature for 30 min. The freshly prepared enhanced chemiluminescence (ECL) (180–501, Tanon, China) mixed solution (A: B = 1:1) was added dropwise to the protein side of the membrane and exposed in the dark room.

### 2.6 Knockdown and overexpression of MFN2 in cells

The *in vitro* cell model of PD was constructed by treating SH-SY5Y cells (iCell-h187, iCell Bioscience Inc., China) with 1-methyl-4-phenylpyridinium (MPP^+^) (HY-W008719, MCE, USA). To further explore the function of MFN2 gene in SH-SY5Y cells, pcDNA3.1-EGFP overexpression plasmid carrying MFN2 gene and siRNA plasmid targeting MFN2 were transfected into PD model cells with Lipofectamine 2000 (11668–019, Invitrogen, USA) to achieve overexpression and knockdown of MFN2 gene, respectively. Subsequent experiments were performed 48 h after transfection. CCK-8 Cell Counting Kit (A311-02, Vazyme, China) was used to detect cell viability. The cell suspension was incubated with CCK-8 solution at 37 °C for 1–4 h, and the optical density (OD) values at 450 nm were measured using a microplate reader. Based on the apoptosis detection kit (A211-02, Vazyme, China), the apoptosis of the cells was detected by flow cytometry. After cell transfection, the medium was removed, and cells were washed once with PBS. The cells were digested with trypsin, then cell culture solution was added and cells were gently dispersed. Cells were collected by centrifugation for 5 min and the supernatant was removed. Subsequently, the cells were resuspended in PBS and stained with Annexin V-FITC/PI for flow cytometry analysis.

In addition, levels of inflammatory and oxidative stress factors were measured in different groups using human interleukin 1β (IL-1β) ELISA kit (YJ883963, mlbio, China), human interleukin 6 (IL-6) ELISA kit (YJ998529, mlbio, China), human tumor necrosis factor alpha (TNF-*α*) ELISA kit (YJ2233699, mlbio, China), human reactive oxygen species (ROS) ELISA kit (YJ330023, mlbio, China), human malondialdehyde (MDA) ELISA kit (YJ984588, mlbio, China), human glutathione/oxidized glutathione (GSH/GSSG) ELISA kit (YJ588963, mlbio, China) and human 4-Hydroxynonenal (4-HNE) ELISA kit (YJ442566, mlbio, China) according to the manufacturer’s protocol. It is worth noting that the kits used for ELISA detection based on mouse samples were different from ELISA detection based on cell samples. Specifically, mouse IL-1β ELISA kit (YJ301814, mlbio, China), mouse IL-6 ELISA kit (YJ34291, mlbio, China), mouse TNF-*α* ELISA kit (YJ002095, mlbio, China), mouse ROS ELISA kit (YJ556999, mlbio, China), mouse MDA ELISA kit (YJ524889, mlbio, China), mouse GSH/GSSG ELISA kit (YJ695588, mlbio, China) and mouse 4-HNE ELISA kit (YJ965545, mlbio, China).

### 2.7 Nissl staining detection

The sample tissues fixed in 4% paraformaldehyde were dehydrated in different concentrations (50, 75, 80, 90, 95, and 100%) of ethanol. The dehydrated tissue samples were immersed in xylene for 30 min each time and repeated twice. Then, immerse the tissue in melted paraffin, maintain a constant temperature of 60 °C, and immerse in wax twice for 1 h each time. Prepare the embedding frame, put the melted wax into the Prepare the embedding frame, put the tissue soaked in wax into the embedding box before the wax solidification, and trim the wax block after cooling and solidification. Install the repaired wax block onto the metal wax holder, and the thickness of the paraffin slice is generally 4–6 μm. Using ophthalmic tweezers, gently lay the wax sheet flat on a water surface at 40–45 °C, and use the tension and temperature of the water to naturally flatten the slightly wrinkled wax sheet. After the slice is fully flattened on a constant temperature water surface, the wax slide is scooped out to the middle of the glass slide and the remaining water on the slide is poured out. The slide is then placed in a constant temperature oven at 60–65 °C for baking. After the sections are sequentially dewaxed with xylene, soaked in ethanol of different concentrations (90 and 70%) and distilled water, they are stained with Nissl staining solution (C0117, Beyotime, China). Then, they are rinsed with running water and treated with ethanol of specific concentrations (95%) and xylene. Finally, seal the slices with neutral gum and observe, photograph, and analyze the pathological section status under a microscope.

### 2.8 Morphological observation of subcellular structure under electron microscope

The samples fixed in glutaraldehyde were taken out and cut into rectangular tissue blocks of 1.5 mm*2.0 mm in size. Then, rinsed with PBS buffer 3 times, osmium tetroxide fixed for 2 h, 50–100% ethanol dehydrated step by step, 100% acetone dehydrated twice. Subsequently, the sample was infiltrated with EPON812 for 2 h and embedded. The embedded samples were cut into 1 mm thick semi-thin sections for light microscopy localization. Subsequently, ultra-thin sections were prepared and stained with conventional uranyl acetate and lead citrate. Finally, the ultrastructure of the brain tissue was observed under a transmission electron microscope.

### 2.9 Statistical analysis

All statistical analyses were performed in the GraphPad Prism 8.0 package. Continuous variables were analyzed using the standard deviation of mean (SEM). The differences between the two groups were analyzed using two-tailed t-test, while the differences among multiple groups were analyzed by one-way analysis of variance (ANOVA) and Tukey’s test. To reduce experimental errors and mitigate the influence of random occurrences on results, an experiment typically requires at least 3 repetitions. The *p* < 0.05 was considered statistically significant.

## 3 Results

### 3.1 Expression levels of DHODH, MFN1, MFN2, GPX4 and FSP1 in PD model of mice

**Figure 1:**
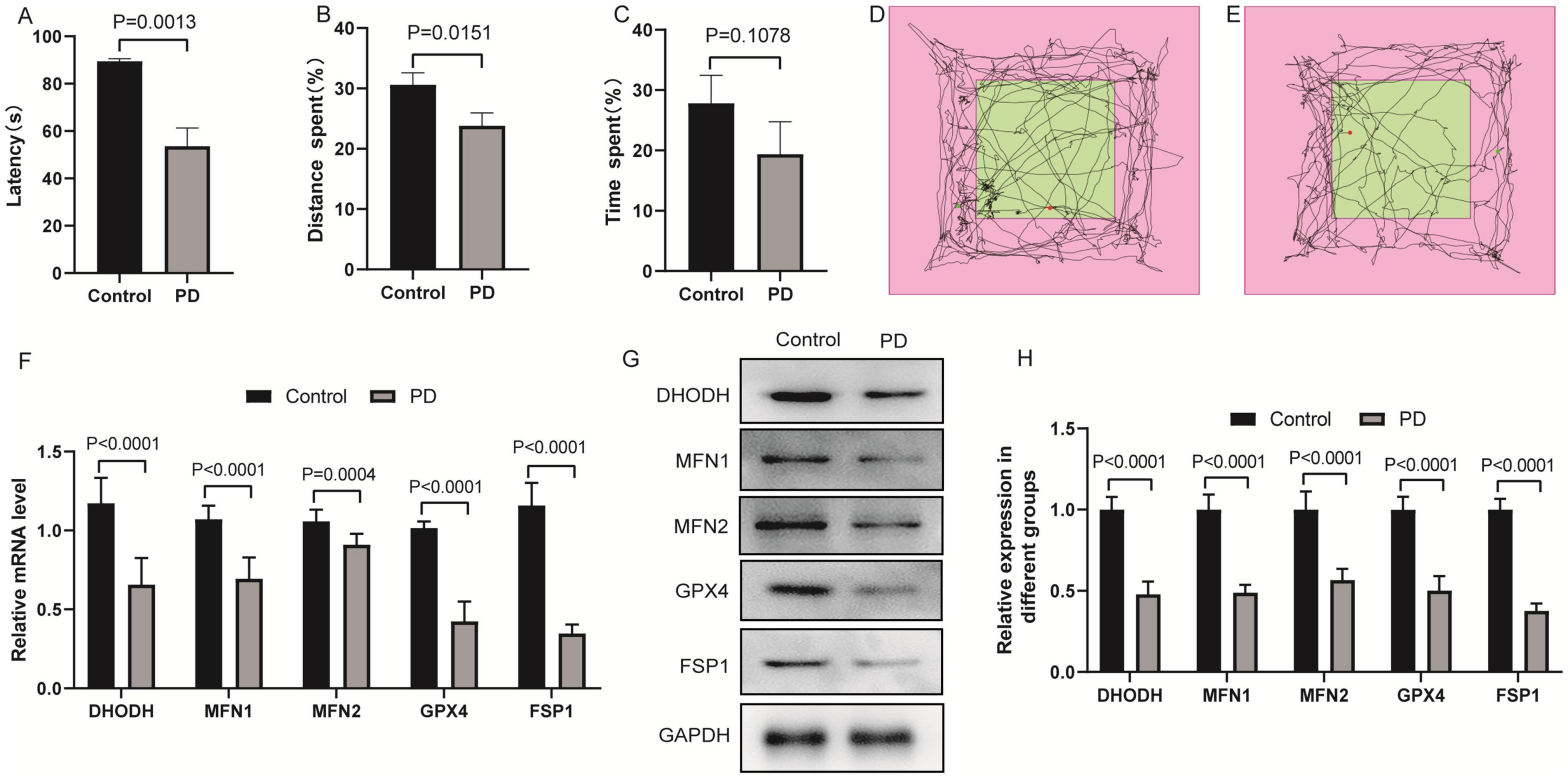
Expression levels of DHODH, MFN1, MFN2, GPX4 and FSP1 in PD model of mice. **(A)** Statistical analysis of the fall latency of mice on the rotarod; **(B)** Percentage of distance traveled in the target area in the open field test; **(C)** Percentage of time spent in the target area in the open field test; **(D)** The trajectory diagram of the open field test for the control group; **(E)** The trajectory diagram of the open field test for the PD group; **(F)** Detection of DHODH, MFN1, MFN2, GPX4 and FSP1 expression in PD group by qRT-PCR; **(G)** The expression bands of DHODH, MFN1, MFN2, GPX4 and FSP1 proteins in control and PD groups were detected by western blotting; **(H)** The relative expression levels of DHODH, MFN1, MFN2, GPX4 and FSP1 protein in control and PD groups. The differences between the two groups were analyzed using two-tailed t-test.

Mitochondrial dysfunction and ferroptosis are key pathological features of PD. Dihydroorotate dehydrogenase (DHODH), MFN1, MFN2, glutathione peroxidase 4 (GPX4), and ferroptosis suppressor protein 1 (FSP1) are involved in mitochondrial metabolism, mitochondrial fusion, and ferroptosis regulation, respectively. To explore whether DHODH, MFN1, MFN2, GPX4, and FSP1 are involved in PD pathogenesis, we investigated their expression patterns in mice PD model. C57BL/6 J mice were used to establish the PD model. Motor coordination was assessed by rotarod test, and self-cognitive behavior and exploration ability were evaluated by open field test. Additionally, qRT-PCR and western blot were used to detect the mRNA and protein levels of DHODH, MFN1, MFN2, GPX4, and FSP1. The results of the rotarod test showed that the motor coordination ability of the PD group was significantly down-regulated compared with the control group (Figure 1A). Open field test showed that the distance and time spent in the target area of the PD group decreased, suggesting that the self-cognitive behavior and exploration ability of the mice were down-regulated (Figures 1B–E). The results of qRT-PCR (Figure 1F) and western blot (Figures 1G,H) showed that DHODH, MFN1, MFN2, GPX4 and FSP1 were significantly down-regulated in the brain tissue of PD mice. These results indicate that PD mice exhibit motor and cognitive deficits accompanied by down-regulation of DHODH, MFN1, MFN2, GPX4, and FSP1, suggesting these molecules may be associated with PD progression. Among them, MFN2 was selected for subsequent studies due to its critical role in maintaining mitochondrial fusion and function.

### 3.2 Effect of MFN2 on cell viability and apoptosis

**Figure 2:**
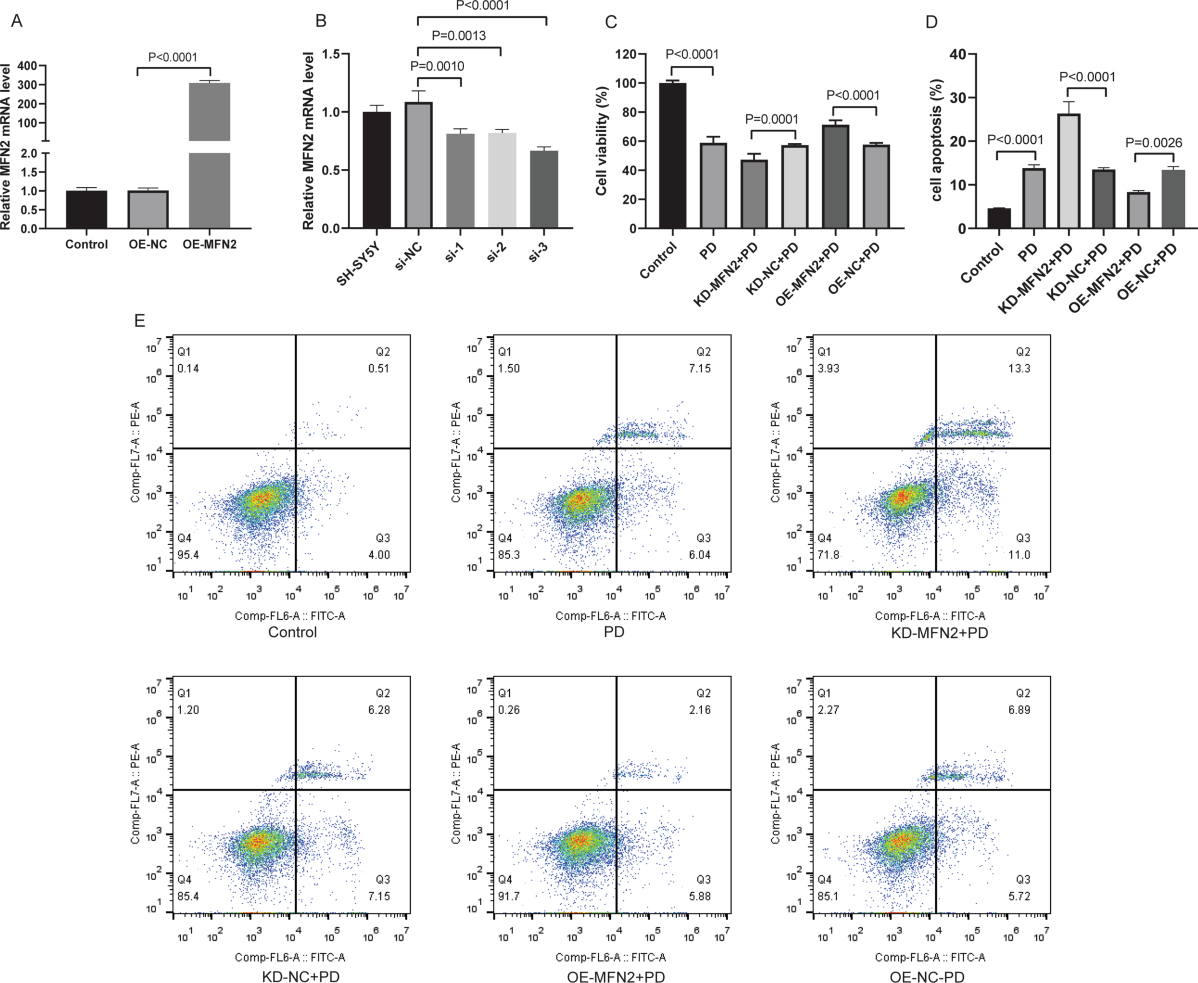
Up-regulation of MFN2 expression increased cell viability and inhibited apoptosis. **(A)** The expression of MFN2 overexpressed plasmid was detected by qRT-PCR; **(B)** The knockdown effect of 3 interference plasmids on MFN2 was detected by qRT-PCR; **(C)** Cell viability was detected by CCK-8 assay; **(D)** Statistical histogram of apoptosis in flow cytometry; **(E)** Flow cytometry was used to detect apoptosis. Control, normal cell group; PD, PD model cell group; KD-MFN2 + PD, MFN2 knockdown in PD model cell group; KD-NC + PD, control group for MFN2 knockdown in PD model cell; OE-MFN2 + PD, MFN2 overexpression in PD model cell group; OE-NC + PD, control group for MFN2 overexpression in PD model cell. The differences among multiple groups were analyzed by one-way analysis of variance (ANOVA) and Tukey’s test.

Based on the *in vivo* findings that MFN2 is down-regulated in PD mice, we further explored its function in cellular model to clarify its direct effects on cell, as cell models allow precise control of experimental variables. SH-SY5Y cells were treated with MPP^+^ to establish *in vitro* PD model. MFN2 overexpression plasmid or interfering plasmid was transfected into the PD model cells. Cell viability was detected by CCK-8 assay, and apoptosis was analyzed by flow cytometry. The overexpression effect of MFN2 in the overexpression plasmid was detected by qRT-PCR, and the results showed that the expression level of MFN2 was significantly increased (Figure 2A). In addition, qRT-PCR results showed that the knockdown effect of interfering plasmid 3 (si-3) was the best (Figure 2B). Therefore, si-3 was selected for subsequent knockdown experiments. The plasmid was transfected into PD model cells. The results of CCK-8 assay showed that the cell viability in the PD group was decreased. After treatment with MFN2-siRNA for interference, the cell viability further declines. However, after transfection with the MFN2 overexpression plasmid, the cell viability significantly increases (Figure 2C). Subsequently, the apoptosis of each group was detected by flow cytometry (Figures 2D,E). Compared with the control group, the apoptosis of PD group was increased. After treatment with MFN2-siRNA for interference, the degree of apoptosis of PD model cells was further up-regulated. However, after transfection with the MFN2 overexpression plasmid, the degree of apoptosis of PD model cells was inhibited. These results indicate that MFN2 regulates cell viability and apoptosis in the PD cell model, suggesting a protective role of MFN2 in maintaining neuronal survival.

### 3.3 Effects of MFN2 on inflammatory factors, oxidative stress factors, ferroptosis and mitochondrial-related factors

**Figure 3:**
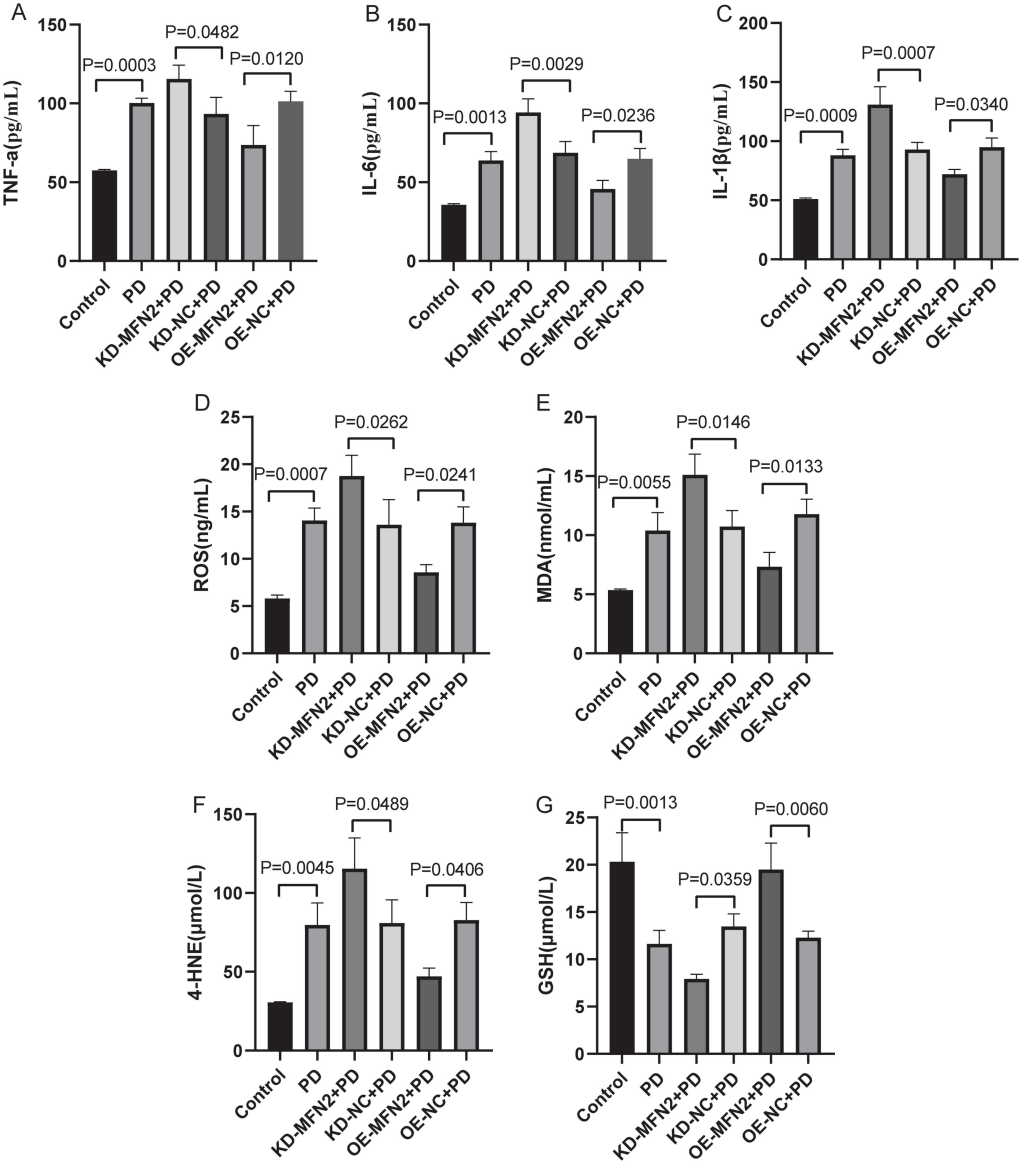
The expression levels of inflammatory and oxidative stress factors in each group were detected by ELISA. **(A–C)** The expression levels of TNF-*α*, IL-6 and IL1-*β* in each group were detected by ELISA; **(D–G)** THE expression levels of ROS, MDA, 4-HNE and GSH in each group were detected by ELISA. Control, normal cell group; PD, PD model cell group; KD-MFN2 + PD, MFN2 knockdown in PD model cell group; KD-NC + PD, control group for MFN2 knockdown in PD model cell; OE-MFN2 + PD, MFN2 overexpression in PD model cell group; OE-NC + PD, control group for MFN2 overexpression in PD model cell. The differences among multiple groups were analyzed by one-way analysis of variance (ANOVA) and Tukey’s test.

**Figure 4:**
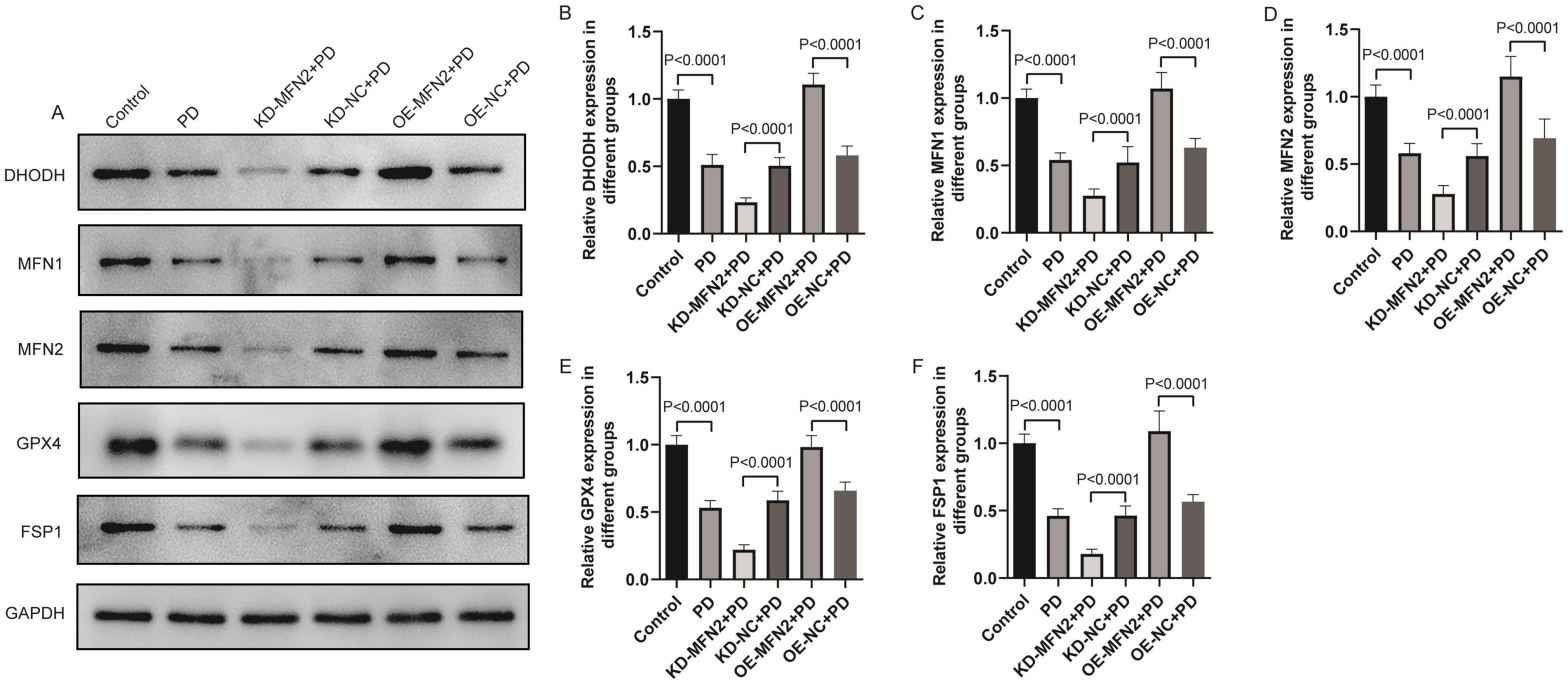
The effects of MFN2 on the expression levels of DHODH, MFN1, GPX4 and FSP1 were detected by western blot. **(A)** The expression bands of DHODH, MFN1, MFN2, GPX4 and FSP1 proteins; **(B–F)** The relative expression levels of DHODH, MFN1, MFN2, GPX4 and FSP1 protein. Control, normal cell group; PD, PD model cell group; KD-MFN2 + PD, MFN2 knockdown in PD model cell group; KD-NC + PD, control group for MFN2 knockdown in PD model cell; OE-MFN2 + PD, MFN2 overexpression in PD model cell group; OE-NC + PD, control group for MFN2 overexpression in PD model cell. The differences among multiple groups were analyzed by one-way analysis of variance (ANOVA) and Tukey’s test.

Inflammation, oxidative stress, ferroptosis, and mitochondrial dysfunction are closely linked to PD pathogenesis. This section aims to investigate the effects of MFN2 on inflammation, oxidative stress, ferroptosis, and mitochondrial function-related factors through experiments in PD cell models. ELISA was used to detect inflammatory factors (TNF-α, IL-6, IL-1β) and oxidative stress factors (ROS, MDA, 4-HNE, GSH). Western blot was performed to measure the protein levels of DHODH, MFN1, GPX4, and FSP1. The results of ELISA showed that the expression levels of TNF-α, IL-6 and IL1-β were up-regulated in PD group. After treatment with MFN2-siRNA for interference, the expression levels of inflammatory factors continued to be up-regulated. However, after transfection with the MFN2 overexpression plasmid, the expression levels of inflammatory factors were down-regulated (Figures 3A–C). The ELISA results also showed that the expression levels of ROS, MDA and 4-HNE were up-regulated in the PD group, while the expression level of GSH was down-regulated, indicating severe oxidative stress response in the PD group. After treatment with MFN2-siRNA for interference, the oxidative stress response was further aggravated. However, after transfection with the MFN2 overexpression plasmid, the oxidative stress response was improved (Figures 3D–G). The results of western blot showed that the expression levels of DHODH, MFN1, MFN2, GPX4 and FSP1 were down-regulated in PD group. After treatment with MFN2-siRNA for interference, the expression levels of DHODH, MFN1, GPX4 and FSP1 were further down-regulated. However, after transfection with the MFN2 overexpression plasmid, the expression levels of DHODH, MFN1, GPX4 and FSP1 were up-regulated (Figures 4A–F). MFN2 may alleviate PD-like pathology in cells by inhibiting inflammation and oxidative stress, up-regulating ferroptosis-related protective factors, and by regulating mitochondrial function.

### 3.4 Overexpression of MFN2 improved motor, cognitive and neurological damage in PD mice

**Figure 5:**
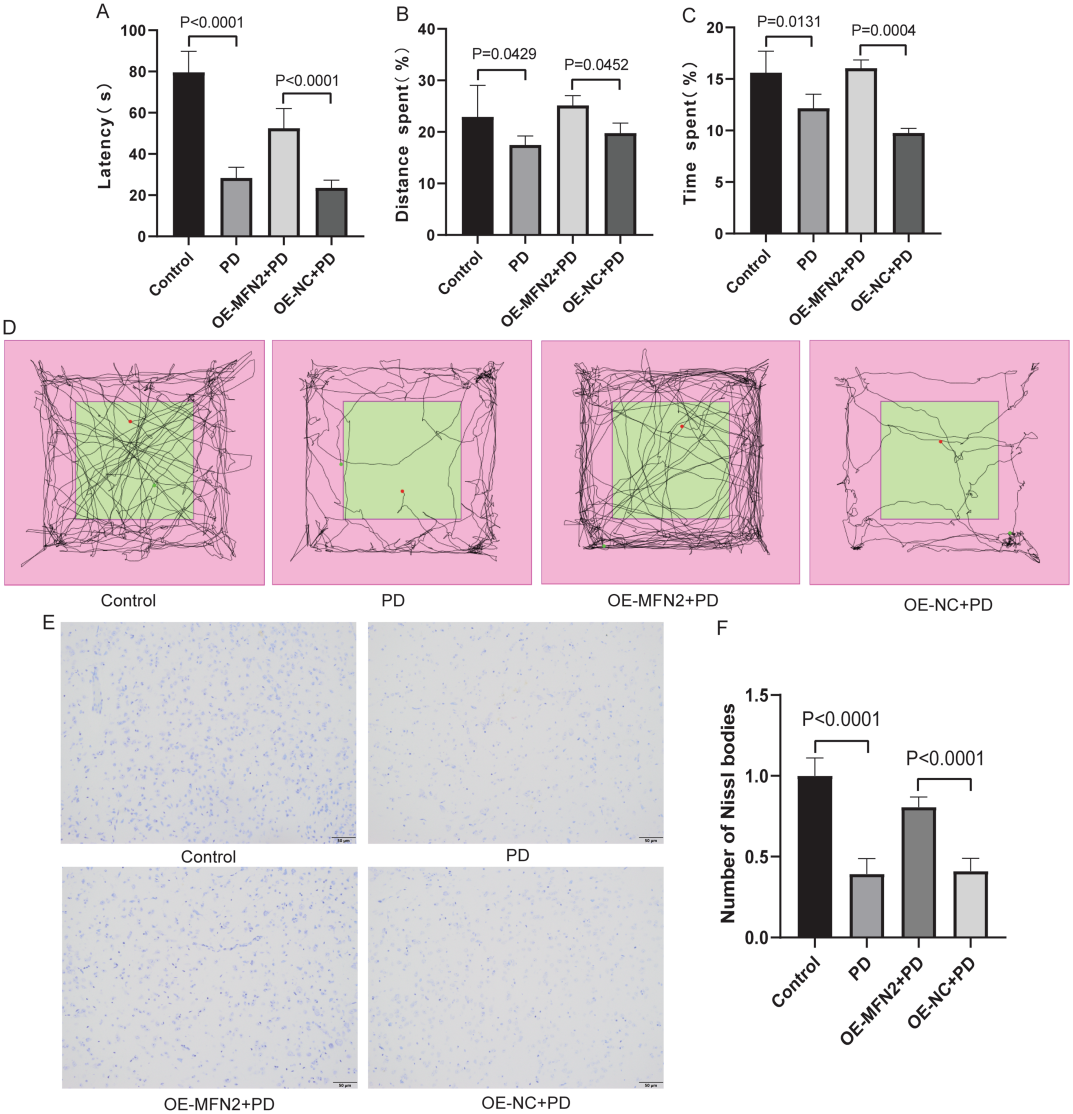
Overexpression of MFN2 improved motor, cognitive and neurological damage in PD mice. **(A)** Statistical analysis of the fall latency of mice on the rotarod; **(B)** Percentage of distance traveled in the target area in the open field test; **(C)** Percentage of time spent in the target area in the open field test; **(D)** The trajectory diagram of the open field test for the control, PD, OE-MFN2 + PD and OE-NC + PD groups; **(E)** Nissl staining image under microscope (200×); **(F)** Statistical histogram of Nissl bodies. Control, Normal mice group; PD, PD model mice group; OE-MFN2 + PD, MFN2 overexpression in PD model mice group; OE-NC + PD, control group for MFN2 overexpression in PD model mice. The differences among multiple groups were analyzed by one-way analysis of variance (ANOVA) and Tukey’s test.

To validate the cellular findings *in vivo*, we explored whether MFN2 overexpression ameliorates PD phenotypes in mice. MFN2 overexpression vector was delivered to PD model mice. Motor coordination was assessed by rotarod test, and self-cognitive behavior and exploration ability were evaluated by open field test. Nissl staining was performed to assess neuronal damage. The results of the rotarod test showed that the motor coordination ability of the PD group was decreased. However, after overexpression of MFN2, the motor ability of the mice was restored to a certain extent (Figure 5A). Open field test showed that the distance and time spent in the target area of the PD group decreased. However, after overexpression of MFN2, the distance and time spent in the target area increased, suggesting that the self-cognition and exploration ability of PD mice had a certain recovery after overexpression of MFN2 (Figures 5B–D). In addition, Nissl staining results showed that the number of Nissl bodies in the brain tissue of PD mice were reduced, suggesting that neuronal function was impaired. After overexpression of MFN2, the number of Nissl bodies increased, suggesting that neuronal function was restored to a certain extent (Figures 5E,F). MFN2 overexpression improves motor and cognitive deficits and reduces neuronal damage in PD mice, confirming its protective role *in vivo*.

### 3.5 Morphological observation of subcellular structure

**Figure 6:**
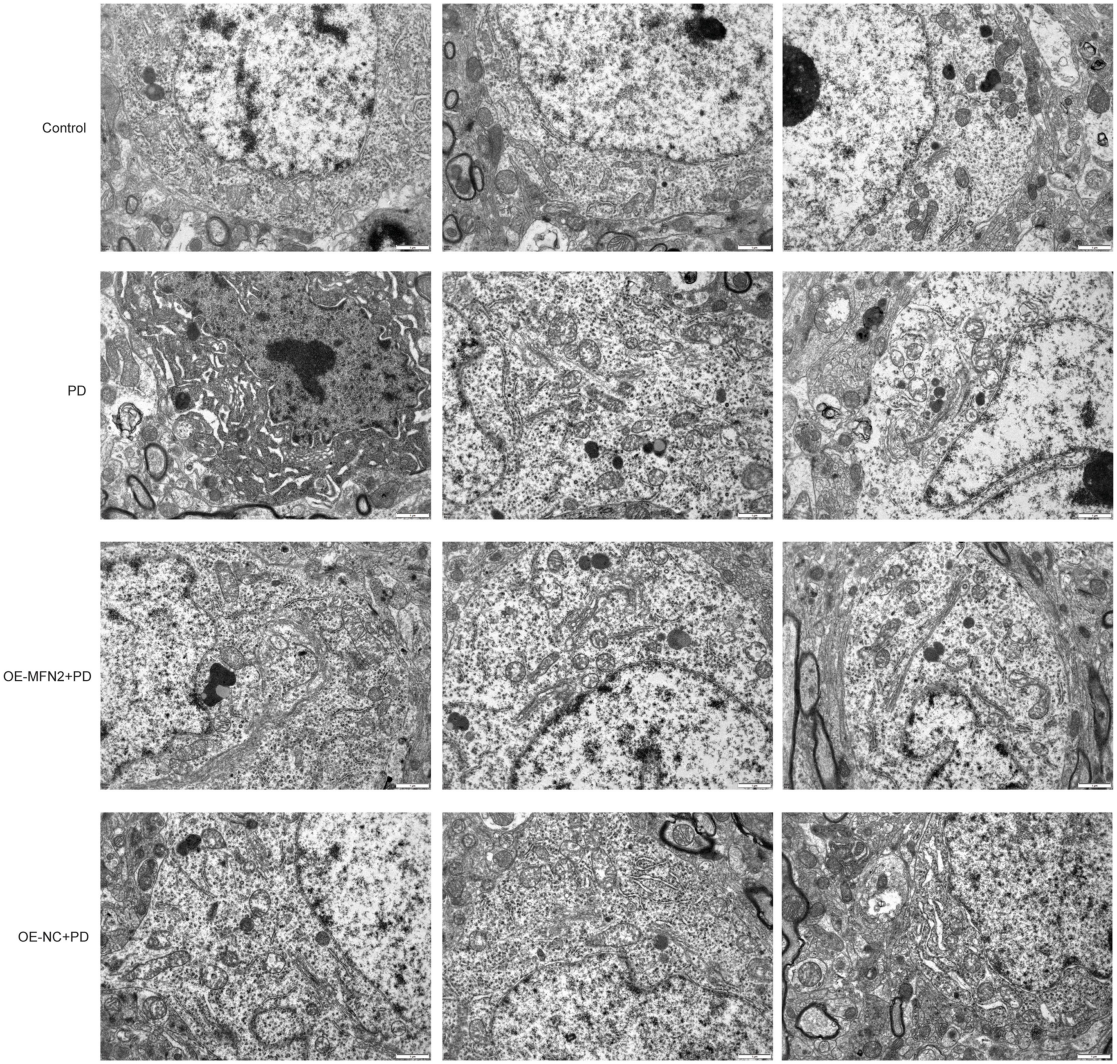
Morphological observation of subcellular structure under electron microscope. Control, Normal mice group; PD, PD model mice group; OE-MFN2 + PD, MFN2 overexpression in PD model mice group; OE-NC + PD, control group for MFN2 overexpression in PD model mice.

Given that MFN2 plays a crucial role in maintaining mitochondrial fusion and function, this section aims to explore whether MFN2 overexpression can ameliorate subcellular morphological abnormalities, particularly those in mitochondrial structure, in PD models. Transmission electron microscopy was used to observe subcellular structures. In the control group, the cell structure was complete and the matrix was evenly distributed. The mitochondrial structure was normal, and the mitochondrial cristae were clear and obvious without breakage or degradation. The mitochondrial matrix was normal without degradation. No autophagy occurred in the cytoplasm (Figure 6). In the PD group, the cell structure was complete and the matrix was evenly distributed. Mitochondrial cristae were breakage and degradation. Mitochondrial matrix was moderately degraded and mitochondria were swollen. No autophagic vesicles or autophagosomes were observed in the cytoplasm. In the OE-MFN2 + PD group, the cell structure was complete and the matrix was evenly distributed. Mitochondrial cristae breakage and degradation were alleviated. Mitochondrial matrix was moderately degraded and mitochondria were swollen. Autophagic vesicles and autolysosomes appeared in the cytoplasm. In the OE-NC + PD group, the cell structure was complete and the matrix was evenly distributed. Mitochondrial cristae were breakage and degradation. Mitochondrial matrix was moderately degraded and mitochondria were swollen. No autophagic vesicles or autophagosomes were observed in the cytoplasm. The above results indicate that MFN2 overexpression alleviates mitochondrial structural damage in the context of PD.

### 3.6 Effects of MFN2 overexpression on inflammatory factors, oxidative stress factors, ferroptosis and mitochondrial-related factors in PD mice

**Figure 7:**
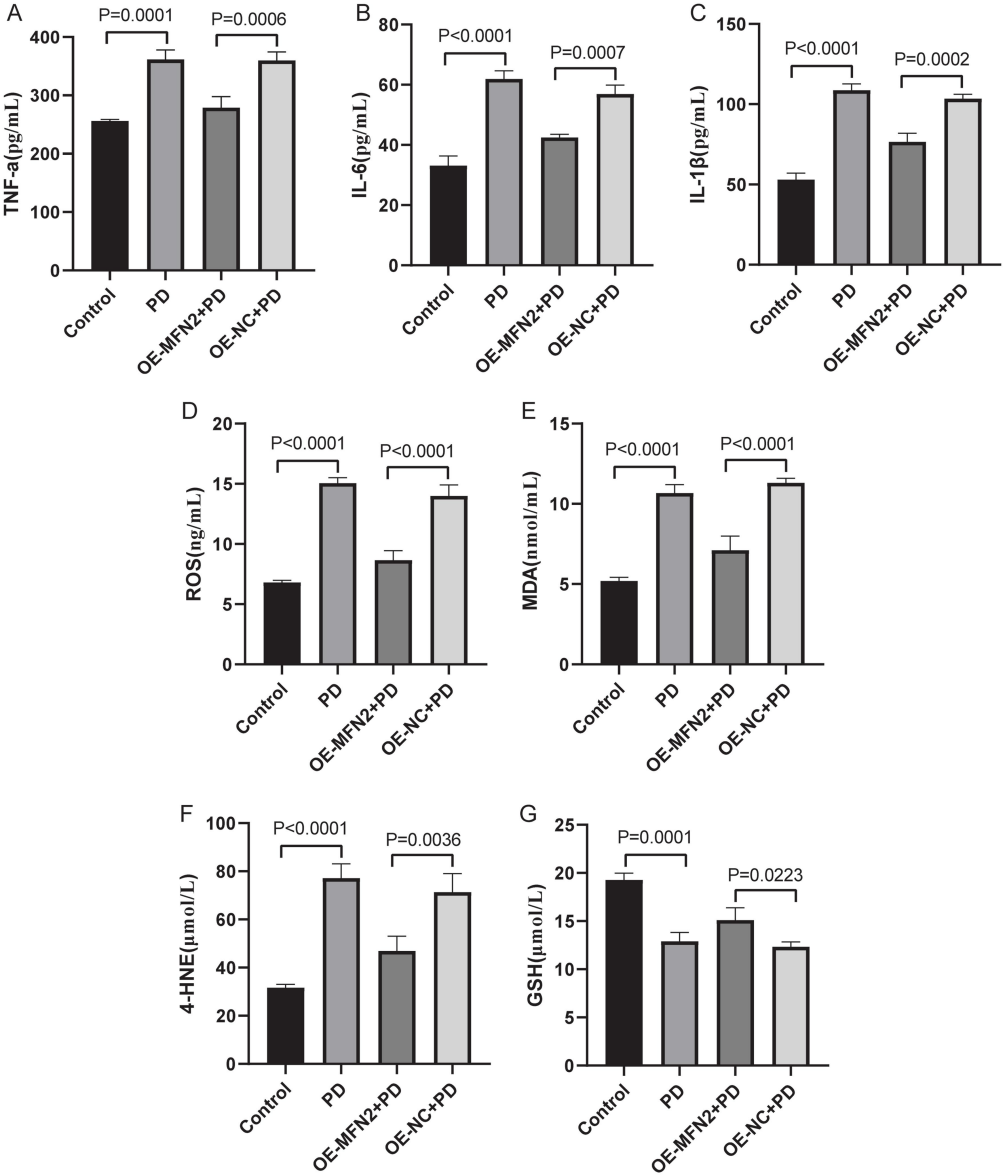
The expression levels of inflammation and oxidative stress factors in brain tissue were detected by ELISA. **(A–C)** The expression levels of TNF-α, IL-6 and IL1-β in each group were detected by ELISA; **(D–G)** The expression levels of ROS, MDA, 4-HNE and GSH in each group were detected by ELISA. Control, Normal mice group; PD, PD model mice group; OE-MFN2 + PD, MFN2 overexpression in PD model mice group; OE-NC + PD, control group for MFN2 overexpression in PD model mice. The differences among multiple groups were analyzed by one-way analysis of variance (ANOVA) and Tukey’s test.

**Figure 8:**
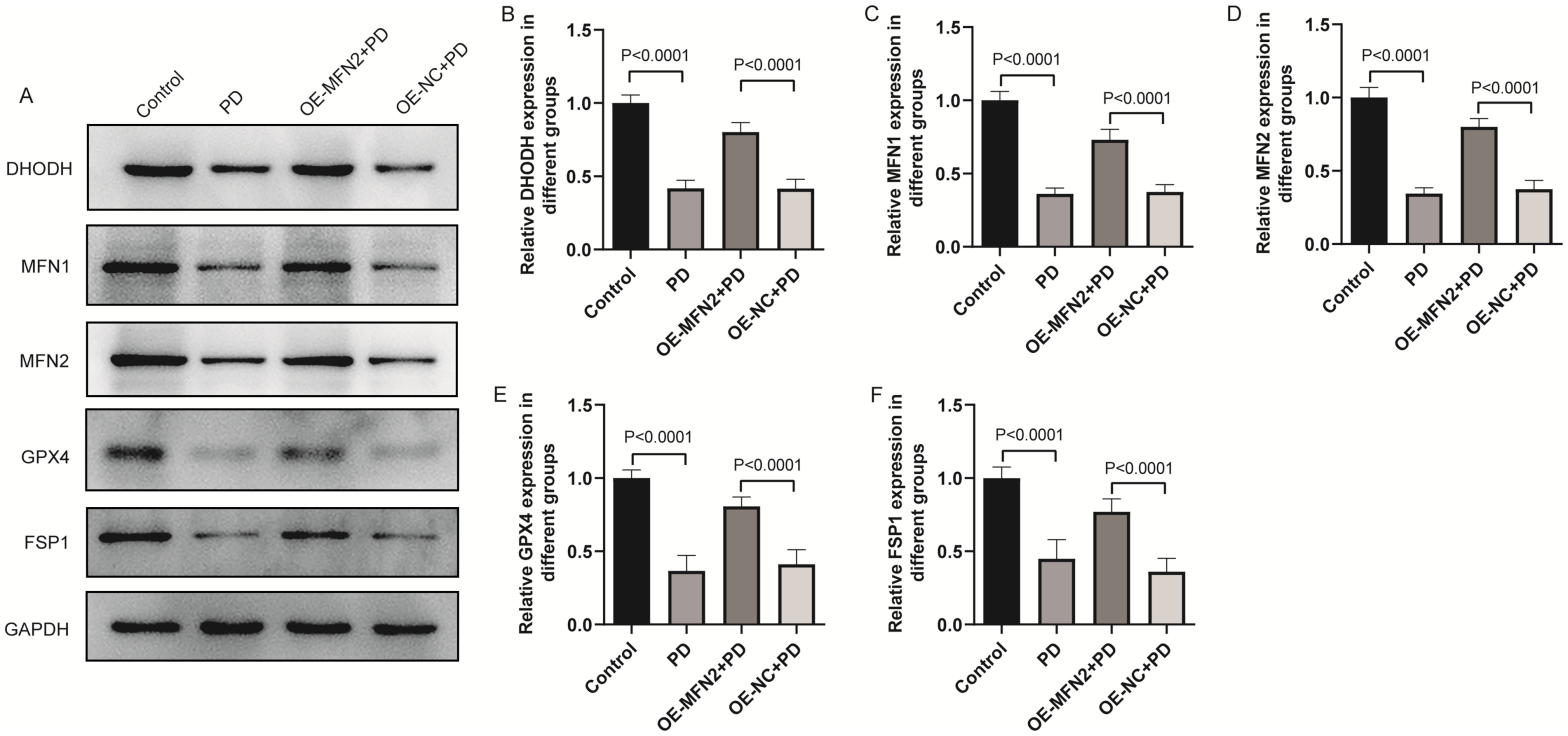
The effect of overexpression of MFN2 on the expression levels of DHODH, MFN1, GPX4 and FSP1 in brain tissue was detected by western blot. **(A)** The expression bands of DHODH, MFN1, MFN2, GPX4 and FSP1 proteins; **(B–F)** The relative expression levels of DHODH, MFN1, MFN2, GPX4 and FSP1 protein. Control, Normal mice group; PD, PD model mice group; OE-MFN2 + PD, MFN2 overexpression in PD model mice group; OE-NC + PD, control group for MFN2 overexpression in PD model mice. The differences among multiple groups were analyzed by one-way analysis of variance (ANOVA) and Tukey’s test.

To confirm consistency between *in vitro* and *in vivo* results, we investigated whether MFN2 overexpression can regulate inflammation, oxidative stress, ferroptosis and mitochondrial function-related factors in PD mice. ELISA was used to detect inflammatory factors (TNF-*α*, IL-6, IL-1*β*) and oxidative stress factors (ROS, MDA, 4-HNE, GSH). Western blot was performed to measure the protein levels of DHODH, MFN1, GPX4, and FSP1. The results of ELISA showed that the expression levels of TNF-α, IL-6 and IL1-β were up-regulated in the brain tissue of PD mice. After overexpression of MFN2, the expression levels of inflammatory factors were down-regulated in in the brain tissue of PD mice (Figures 7A–C). The ELISA results also showed that the expression levels of ROS, MDA and 4-HNE were up-regulated in the brain tissue of PD mice, while the expression level of GSH was down-regulated. After overexpression of MFN2, the expression levels of ROS, MDA and 4-HNE were down-regulated in the brain tissue of PD mice, while the expression level of GSH was up-regulated (Figures 7D–G). Moreover, the expression trends of inflammatory and oxidative stress factors in peripheral blood (Supplementary Figure S1) were consistent with that in brain tissue. The above results suggest that overexpression of MFN2 may help alleviate inflammation and oxidative stress responses in PD mice. In addition, after overexpression of MFN2, the expression levels of DHODH, MFN1, GPX4 and FSP1 were up-regulated in the brain tissue of PD mice (Figures 8A–F). These research results were consistent with the *in vitro* study findings, further confirming that MFN2 may alleviate PD by inhibiting inflammation and oxidative stress, up-regulating ferroptosis-related protective factors, and by regulating mitochondrial function.

## 4 Discussion

PD continues to pose a significant global health challenge, with its prevalence steadily increasing as the population ages (Hirsch et al., 2016). This progressive neurodegenerative disorder, characterized by the loss of dopaminergic neurons in the substantia nigra pars compacta, leads to a complex array of motor and non-motor symptoms, severely compromising patients’ daily lives (Obeso et al., 2010). Despite extensive research, the underlying pathophysiological mechanisms remain only partially understood. In this study, we investigated the potential molecular mechanisms of MFN2 in PD-related pathophysiological processes, including mitochondrial function, ferroptosis, inflammation and oxidative stress. Our experimental design followed a logical progression of “*in vivo* preliminary observation-*in vitro* mechanism exploration-in vivo validation.” Starting with PD model mice, we first observed abnormal motor/cognitive and altered expression of key molecules such as MFN2. Then, we switched to MPP^+^-treated SH-SY5Y cells to elucidate the direct regulation of MFN2 on cell viability, inflammation, oxidative stress, mitochondria and ferroptosis. Finally, we returned to the mouse models to confirm that overexpression of MFN2 improved pathological symptoms and modulates molecular expression consistently with in vitro findings. This design not only ensures the depth of the research but also guarantees the reliability of the conclusions. This study deepened our understanding of PD pathophysiology by identifying MFN2 as a multifunctional regulator that coordinates mitochondrial function, ferroptosis, inflammation, and oxidative stress. Unlike previous studies that focused on the role of MFN2 in mitochondrial fusion alone, we demonstrate that MFN2 simultaneously modulates ferroptosis, oxidative stress, and neuroinflammation in PD models. This multiple regulation highlights that MFN2 is a hub molecule that links mitochondrial dysfunction with other pathogenic mechanisms.

Mitochondrial dysfunction is a hallmark of PD (Moradi Vastegani et al., 2023), and our sub-cellular morphological observations showed abnormal mitochondrial cristae and matrix degradation in the PD group. Overexpression of MFN2 alleviated these mitochondrial abnormalities. MFN2 and MFN1 are both involved in mitochondrial fusion, which is essential for maintaining mitochondrial function (Chen et al., 2003). Defects in MFN2- and MFN1- mediated mitochondrial fusion have been associated with various neurodegenerative diseases, including PD (Navaratnarajah et al., 2021). By promoting mitochondrial fusion, MFN2 and MFN1 can enhance mitochondrial respiration, reduce ROS production, and improve energy metabolism (Chen et al., 2003). Although MFN1 and MFN2 have overlapping functions, they also have distinct roles in mitochondrial dynamics (Chen et al., 2020). In this study, our observation that MFN2 regulates MFN1 expression adds nuance to their functional interplay in PD. Additionally, our also finding that MFN2 modulates DHODH connects mitochondrial dynamics to metabolic pathways. DHODH is an enzyme critical for pyrimidine synthesis and mitochondrial respiration (Barnes et al., 1993), and this relationship has rarely been explored in PD. Ferroptosis has emerged as a crucial mechanism in PD (Wang et al., 2022), and our results showed that key ferroptosis-related factors such as GPX4 (Liu et al., 2023) and FSP1 (Li et al., 2023) were regulated by MFN2 in PD. This is consistent with previous studies (Chen et al., 2023; Zhang et al., 2024) suggesting that MFN2 can modulate ferroptosis. Moreover, mitochondria are closely associated with iron metabolism (Gao et al., 2021). Based on this study, we hypothesize that MFN2 may prevent ferroptosis by maintaining mitochondrial function, a mechanism that connects two major pathogenic mechanisms of PD that are typically studied in isolation.

Oxidative stress is a well-established contributor to PD pathogenesis. Excessive ROS production not only inflicts damage on lipids, proteins, and DNA but also sets in motion a cascade of events leading to neuronal apoptosis (Trist et al., 2019). In neurodegenerative diseases, mitochondrial dysfunction impairs ATP production and promotes the production of ROS. The accumulation of ROS further damages mitochondrial DNA, proteins and lipids, resulting in a vicious cycle of oxidative stress and mitochondrial damage (Wen et al., 2025). A study in cardiomyocytes showed that restoration of MFN2 mediated mitochondrial fusion enhanced mitochondrial oxidative metabolism, reduced cell damage/apoptosis, and inhibited mitochondrial-derived oxidative stress (Ding et al., 2022). Neuroinflammation is a prominent feature of PD (Liu and Chen, 2022). In PD, the brain’s innate immune system becomes dysregulated, with activated microglia and astrocytes being the main drivers of this inflammatory response, and the pro-inflammatory factors they release, such as TNF-*α*, IL-6 and IL1-*β*, can be involved in disease progression (Araújo et al., 2022; Isik and Yeman Kiyak, 2023). The activation of Sting signaling in microglia promotes neuronal death and inflammatory response in spinal cord injury, and MFN2 may mediate the activation of this signaling pathway in microglia (Wei et al., 2024). One study showed that MFN2 in neurons contributes to the regulation of neuroinflammation and proposed that neuronal MFN2 may be a mechanistic connector between neuronal mitochondrial dysfunction and neuroinflammation in neurodegeneration (Harland et al., 2020). Our research results showed that regulating MFN2 affected key oxidative stress markers (ROS, MDA, 4-HNE, GSH) and pro-inflammatory cytokines (TNF-α, IL-6, IL-1β) in both *in vivo* and *in vitro* PD models. This suggested that MFN2 is a critical regulator of both oxidative stress and neuroinflammation in PD, and this finding established a mechanistic link between mitochondrial dynamics and two central pathogenic cascades in PD pathogenesis.

In the in vivo experiments, PD mice showed impaired motor coordination, self-cognitive behavior, and exploration ability, as well as reduced Nissl bodies indicating neuronal damage. Overexpression of MFN2 improved these behavioral and neurological outcomes. In addition, cell experiments showed that MFN2 could regulate cell viability and apoptosis. Previous studies have also shown that MFN2 can protect neurons and ameliorate motor deficits in PD (Zhao et al., 2017; Zhao et al., 2021). Moreover, MFN2 can improve cell apoptosis in rotenone-induced PD cell model (Yang et al., 2018). The consistent beneficial effects of MFN2 observed in vivo and in vitro models underscore that MFN2 may be a critical factor in the future development of therapeutic methods for related diseases.

In conclusion, this study showed that MFN2 plays a critical role in the pathophysiological processes related to PD, including mitochondrial function, ferroptosis, inflammation, and oxidative stress. The results of this study suggested that MFN2 might be a potential therapeutic target for PD. However, this study has certain limitation. Due to the initial experimental design focused on the role of MFN2, the effect of MFN1 overexpression in the MFN2 knockdown model has not been directly detected, and the possibility of functional redundancy between the two has not been completely excluded. Although existing studies have suggested that MFN1 and MFN2 have different roles in mitochondrial dynamics, supporting the non-equivalence of their functions, the lack of direct experimental evidence within the model of this study has to some extent affected the rigor of the conclusion, and further research is needed in the future. In addition, although our current results showed that MFN2 is associated with mitochondrial dysfunction, ferroptosis, inflammation, and oxidative stress, the specific molecular cascades have not been fully elucidated, and further research is needed. Moreover, this study has not incorporated clinical data, resulting in a notable lack of clinical evidence. Subsequent research should conduct validation in clinical samples to consolidate the evidence base for MFN2 as a therapeutic target and facilitate its translation into clinical applications.

## Data Availability

The original contributions presented in the study are included in the article/Supplementary material, further inquiries can be directed to the corresponding authors.

## Supplementary material

SUPPLEMENTARY FIGURE S1 The expression levels of inflammatory and oxidative stress factors in peripheral blood were detected by ELISA. **(A–C)** The expression levels of TNF-α, IL-6 and IL1-β in each group were detected by ELISA; **(D–G)** The expression levels of ROS, MDA, 4-HNE and GSH in each group were detected by ELISA. Control, Normal mice group; PD, PD model mice group; OE-MFN2 + PD, MFN2 overexpression in PD model mice group; OE-NC + PD, control group for MFN2 overexpression in PD model mice. The differences among multiple groups were analyzed by one-way analysis of variance (ANOVA) and Tukey’s test.
