# Supplementary figures and images for: MFN2-a multifaceted guardian against Parkinson’s pathophysiology: mitochondria, ferroptosis, inflammation and oxidative stress
Source: Front Aging Neurosci. 2025 Sep 16;17:1611958. doi: 10.3389/fnagi.2025.1611958 (PMC12479552; doi:10.3389/fnagi.2025.1611958)

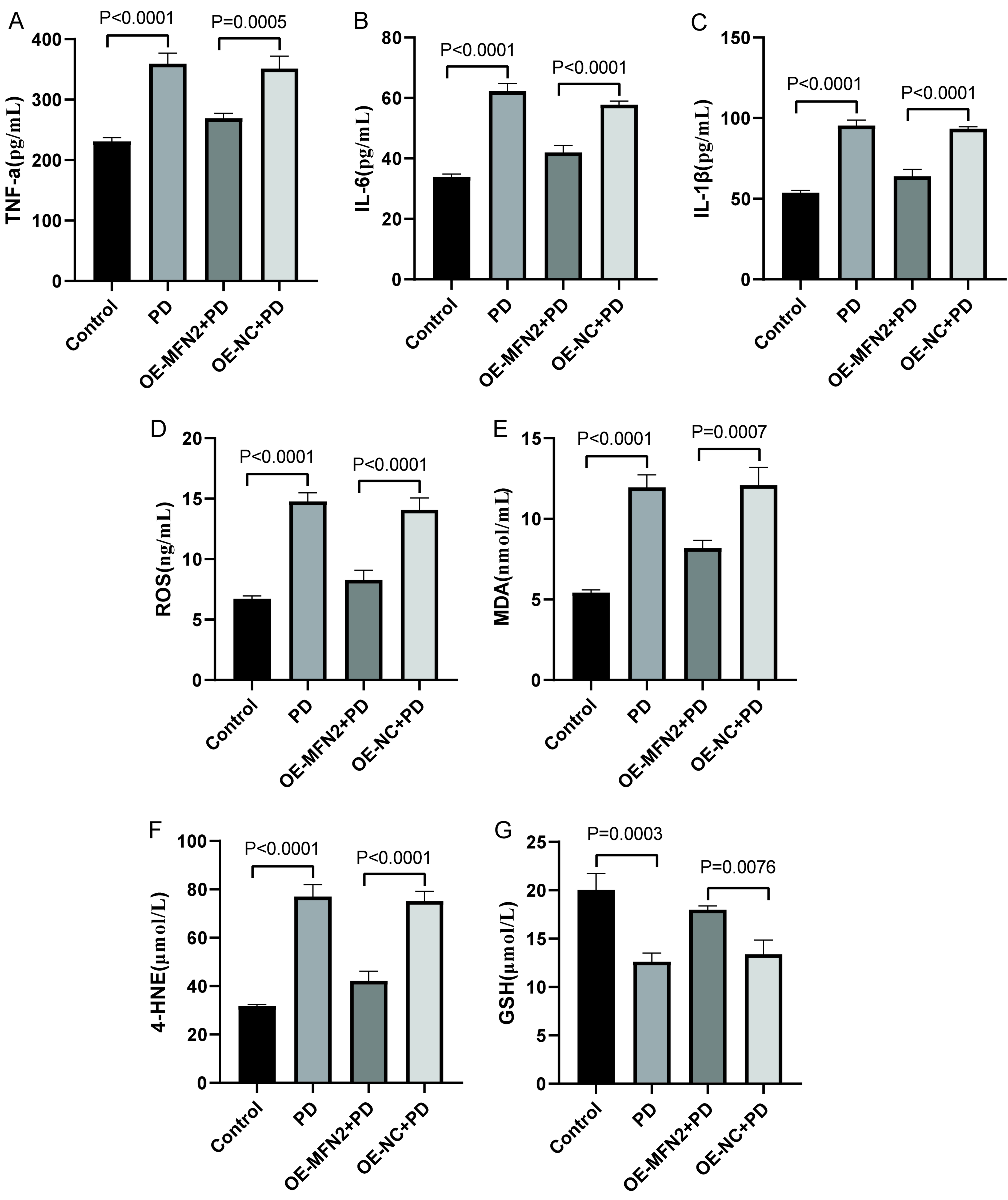

Supplement: SUPPLEMENTARY FIGURE S1 — The expression levels of inflammatory and oxidative stress factors in peripheral blood were detected by ELISA. (A–C) The expression levels of TNF-α, IL-6 and IL1-β in each group were detected by ELISA; (D–G) The expression levels of ROS, MDA, 4-HNE and GSH in each group were detected by ELISA. Control, Normal mice group; PD, PD model mice group; OE-MFN2 + PD, MFN2 overexpression in PD model mice group; OE-NC + PD, control group for MFN2 overexpression in PD model mice. The differences among multiple groups were analyzed by one-way analysis of variance (ANOVA) and Tukey’s test. [file Image_1.tif]
