# Supplementary material for: MFN2-a multifaceted guardian against Parkinson’s pathophysiology: mitochondria, ferroptosis, inflammation and oxidative stress
Source: Front Aging Neurosci. 2025 Sep 16;17:1611958. doi: 10.3389/fnagi.2025.1611958 (PMC12479552; doi:10.3389/fnagi.2025.1611958)
Supplement: Supplementary file 2 [file Table_1.docx]

Table S1 All primer sequences

| Mice | Primer name | Primer sequence (5' to 3') |
| --- | --- | --- |
|  | β-actin-F | TGTTACCAACTGGGACGACA |
|  | β-actin-R | CTGGGTCATCTTTTCACGGT |
|  | DHODH-F | GAGAGTCCTGGGCCATAAATTC |
|  | DHODH-R | CACACTTCCCACCTCAACAA |
|  | MFN1-F | ACAGTGGGCTGGAAACTAATC |
|  | MFN1-R | GCTGCTTAAACGCTCTCTCT |
|  | MFN2-F | CAAGACCGGCTGAGGTTTATT |
|  | MFN2-R | CCTTTCCACTTCCTCCGTAATC |
|  | GPX4-F | CCGATATGCTGAGTGTGGTTTA |
|  | GPX4-R | GGCTGCAAACTCCTTGATTTC |
|  | FSP1-F | CCTTGCCCTTCTCACATCTTATC |
|  | FSP1-R | CTGCTTCACCATGTCCTCATAG |
| Cell | β-actin-F | GGAAATCGTGCGTGACATTAAG |
|  | β-actin-R | AGCTCGTAGCTCTTCTCCA |
|  | MFN2-F | GGACGATTACCAGATGGACTTC |
|  | MFN2-R | ACCCAGTCCTTCCTCTATGT |
